# The Danish Aging and Cognition (DanACo) cohort

**DOI:** 10.1186/s12877-024-04841-5

**Published:** 2024-03-07

**Authors:** Marie Grønkjær, Erik Lykke Mortensen, Cathrine Lawaetz Wimmelmann, Trine Flensborg-Madsen, Merete Osler, Gunhild Tidemann Okholm

**Affiliations:** 1 Center for Clinical Research and Prevention, Copenhagen University Hospital – Bispebjerg and Frederiksberg, Nordre Fasanvej 57, 2000 Frederiksberg, Denmark; 2 Department of Public Health, Unit of Medical Psychology, Section of Environmental Health, University of Copenhagen, Øster Farimagsgade 5, Copenhagen, 1353 Denmark; 3 Center for Healthy Aging, University of Copenhagen, Blegdamsvej 3B, 2200 Copenhagen N, Denmark; 4 Centre for Childhood Health, Islands Brygge 41, 2300 Copenhagen S, Denmark; 5 Department of Public Health, Section of Epidemiology, University of Copenhagen, Øster Farimagsgade 5, Copenhagen, 1353 Denmark

**Keywords:** Cognitive decline, Intelligence, Børge Priens Prøve (BPP), Aging, DanACo, LiKO-15, DiaKO-19

## Abstract

**Background:**

With aging populations worldwide, identification of predictors of age-related cognitive decline is becoming increasingly important. The Danish Aging and Cognition Cohort (DanACo) including more than 5000 Danish men was established to investigate predictors of age-related cognitive decline from young adulthood to late mid-life.

**Construction and content:**

The DanACo cohort was established through two separate data collections with identical designs involving a follow-up examination in late mid-life of men for whom intelligence test scores were available from their mandatory conscription board examination. The cohort consists of 5,183 men born from 1949 through 1961, with a mean age of 20.4 years at baseline and a mean age of 64.4 years at follow-up. The baseline measures consisted of height, weight, intelligence test score and educational level collected at the conscription board examination. The follow-up assessment consisted of a re-administration of the same intelligence test and a comprehensive questionnaire covering socio-demographic factors, lifestyle, and health-related factors. The data were collected in test sessions with up to 24 participants per session. Using the unique personal identification number assigned to all Danes, the cohort has been linked to data from national administrative and health registers for prospectively collected data on socioeconomic and health-related factors.

**Utility and discussion:**

The DanACo cohort has some major strengths compared to existing cognitive aging cohorts such as a large sample size (*n* = 5,183 men), a validated global measure of cognitive ability, a long retest interval (mean 44.0 years) and the availability of prospectively collected data from registries as well as comprehensive questionnaire data. The main weakness is the low participation rate (14.3%) and that the cohort consists of men only.

**Conclusion:**

Cognitive decline is a result of a summary of factors across the life-course. The DanACo cohort is characterized by a long retest interval and contains data on a wealth of factors across adult life which is essential to establish evidence on predictors of cognitive decline. Moreover, the size of the cohort ensures sufficient statistical power to identify even relatively weak predictors of cognitive decline.

**Supplementary Information:**

The online version contains supplementary material available at 10.1186/s12877-024-04841-5.

## Background

With aging populations worldwide, identification of predictors of individual differences in age-related cognitive decline is becoming increasingly important [1]. However, it has proved difficult to identify consistent predictors of cognitive decline [2, 3]. Cognitive functioning in old age is affected by a multitude of factors across the life-course. Moreover, low intelligence measured in childhood or young adulthood has been associated with several of the medical conditions suspected to accelerate cognitive decline (e.g. hypertension, type 2 diabetes or cardiovascular disease) [4, 5]. Consequently, early life intelligence might to some extent be the driver of the observed associations between various health states and cognitive decline. This highlights the need for baseline measures of cognitive ability to be assessed early in life when investigating predictors of cognitive decline. Moreover, it has been emphasized that a long follow-up is essential for identifying predictors of cognitive decline [6]. Unfortunately, there are very few aging cohorts that include early life measures of cognitive ability [2, 7]. The Lothian Birth Cohorts, founded by Professor Ian Deary and late Professor John Starr, are some of the few existing cohorts including measures of cognitive abilities in early life (age 11 years) and old age (mean age at first follow-up: 69.6 (LBC1946) and 79.1 (LBC1921) years old) [3]. The cohorts have several waves of follow-up and have been pioneering within the field of cognitive aging [3]. However, the cohorts are relatively small with 550 (LBC1921) and 1090 (LBC1946) participants in the first waves of cognitive follow-up.

In Denmark, intelligence testing has been part of the conscription board examinations since 1957, and large conscription databases have been established as an important resource for research on early life intelligence as a predictor of later life health outcomes [8]. The Danish Aging and Cognition Cohort (DanACo) was established through collaboration with the Danish Defence, making it possible to invite men aged 53–73 years and re-administer the military intelligence test to assess cognitive decline from young adulthood to late midlife. The DanACo cohort was established through two separate data collections with an identical design, but with focus on different potential predictors. Despite this difference in the initial focus, analyses have confirmed that the two study samples were comparable on baseline characteristics supporting the pooling of the two study samples (for more details see the paragraph on ‘ Strengths and weaknesses’ in the ‘ Utility and discussion’ section). The DanACo cohort thus consists of 5,183 men who have taken the same intelligence test in young adulthood and in late midlife and contains comprehensive questionnaire- and registry-data on potential predictors of cognitive decline.

## Construction and content

### Design

The DanACo cohort has been established by pooling the study samples of two follow-up studies, namely the Lifestyle and Cognition Follow-up study 2015 (LiKO-15) and the Diabetes and Cognition Follow-up study 2019 (DiaKO-19). The LiKO-15 study focused on the influence of alcohol consumption, alcohol use disorders (AUD) and other psychiatric disorders on cognitive decline, whereas the DiaKO-19 study focused on the influence of type 2 diabetes and depression on cognitive decline. The design of the two studies was identical and was based on a follow-up in late midlife including a re-assessment of the cognitive abilities of Danish men with the military intelligence test. Examination data from Danish conscription boards were used as baseline data and the follow-up assessment consisted of the military intelligence test and a comprehensive questionnaire on sociodemographic factors, lifestyle, and health-related factors. The cohort is unique because the cognitive ability of the participants has been assessed using the same intelligence test in young adulthood (mean age: 20.4 years old) and in late mid-life (mean age: 64.4 years old) allowing for a direct measure of the cognitive changes over a period of more than 40 years.

### Recruitment

The DanACo participants were recruited from existing conscription board databases and were born from 1949 through 1961 (LiKO-15: 1950–1961; DiaKO-19: 1949–1960) and examined at the conscription board from 1967 through 1989 (LiKO-15: 1968–1989; DiaKO-19: 1967–1984). The men invited to participate in LiKO-15 were recruited from two existing databases with conscription board data: a psychiatric database established by Urfer-Parnas et al. [9] and the Danish Conscription Database (DCD) [10]. The psychiatric database was designed to include a large proportion (30.5%) of men with a psychiatric diagnosis from Danish hospitals and was geographically restricted to men examined in the northern part of Zealand. The majority of LiKO-15 participants (78.1%) were recruited among the men with and without prior psychiatric diagnosis from the psychiatric database. The men invited to participate in DiaKO-19 were recruited from the DCD; a database which was designed to cover all Danish men examined by the conscription boards across all of Denmark from 1957 through 1977 [10]. The DCD was linked with Danish hospital registers to exclude men with a hospital diagnosis of type 1 diabetes or with a hospital diagnosis of depression before their conscription board examination. Moreover, men who had participated in LiKO-15 or who following an invitation to LiKO-15 had expressed a wish not to be contacted again were not invited to participate in DiaKO-19.

The common inclusion criteria for the LiKO-15 and DiaKO-19 studies were: 1) available data on intelligence test scores from the conscription board examination and 2) living within 50 km’s of the two locations where the data collection took place. For LiKO-15, it was furthermore a criterium that the men were examined by the conscription board in the eastern part of Denmark, as the psychiatric database exclusively included men examined in this area.

The potential participants were sent an invitation letter with a description of the background for the project and the course of the follow-up examination. Non-responders received a reminder postcard. In LiKO-15 both invitation letters and reminder postcards were sent by regular mail whereas in DiaKO-19 the invitation letter was sent through a secure digital mail service available for all Danish citizens. At the time of the data collection, the proportion subscribing to the digital mail service was 92.3% in the source population for DiaKO-19.

### Number of participants and participation rate

**Fig. 1:**
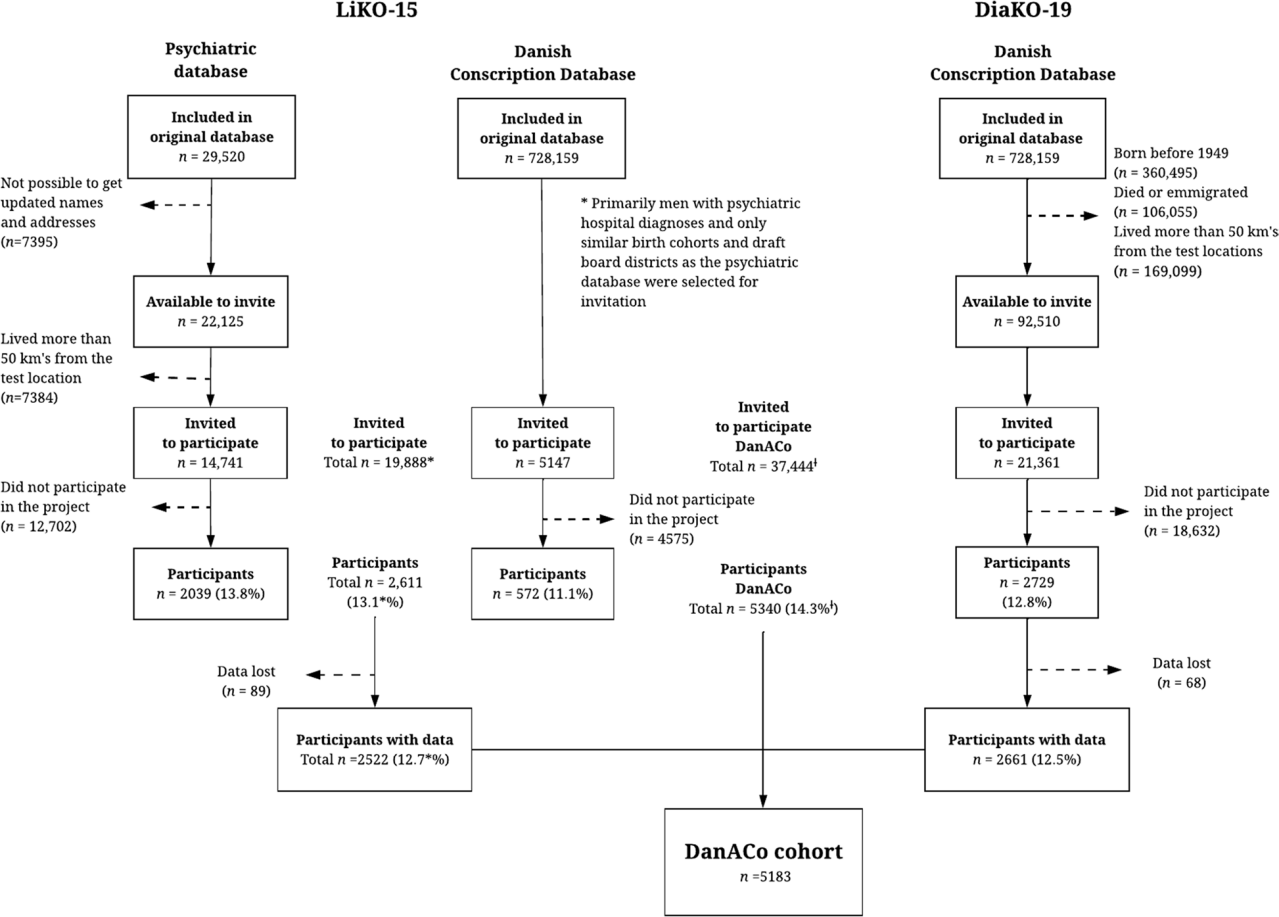
Flow-chart of the DanACo cohort. Abbreviations: LiKO-15: Lifestyle and Cognition Follow-up study 2015; DiaKO-19: Diabetes and Cognition Follow-up study 2019; DanACo: Danish Aging and Cognition. The included percentages were calculated as proportions of the number of men invited to the LiKO-15 and DiaKO-19 studies, respectively. For LiKO-15, the percentages marked with * are calculated based on the total number of participants invited from the Psychiatric database and the Danish Conscription Database. ^†﻿^As 3,805 of the LiKO-15 non-participants were also invited to the DiaKO-19 study, the total number of non-participants in the DanACo cohort, 37,444 men, is equal to the combined number of non-participants from the LiKO-15 and DiaKO-19 studies (35,909) minus the 3,805 participants that were invited twice

The flow of participants from recruitment to participation is presented in Fig. 1. A total of 37,444 men were invited to participate in either the LiKO-15 or DiaKO-19 studies, and 5,340 men participated resulting in an overall participation rate of 14.3% and the participation rate was similar in the two individual studies (LiKO-15: 13.1%; DiaKO19: 12.8%).

In LiKO-15, a total of 19,888 men were invited to participate in the follow-up, and 2,611 participated resulting in a participation rate of 13.1%. Unfortunately, follow-up data from 89 participants were lost due to technical problems with the Military’s computer system leaving a total of 2,522 participants. In DiaKO-19, a total of 21,361 men were invited, of which 3,805 had been invited to but had not participated in the LiKO-15 study. A total of 2,729 men participated in the DiaKO-19 study resulting in a participation rate of 12.8%. Unfortunately, follow-up data was lost for 68 participants due to technical problems with the Military’s computer system leaving a total of 2,661 participants. A total of 5,340 men participated in the LiKO-15 and DiaKO-19 studies and data was lost for 157 men resulting in a combined population of 5,183 men that constitutes the DanACo cohort. For a comparison of baseline characteristics of participants and non-participants see the paragraph on ‘ Generalizability’ in the ‘ Utility and discussion’ section.

### Baseline assessment and measures

The conscription board examinations are considered the baseline assessments of the DanACo cohort and baseline data thus stem from digitized conscription board records. All Danish men are required by law to appear before the conscription board at the age of 18, and although this can be postponed until age 26 years, most men (84.8% among men born 1949–1960) appear at 18–20 years. The examination includes a paper-and-pencil administered intelligence test, an interview, and a physical examination, including anthropometric measurements assessed by a conscription board physician. The conscription board examinations have been described in detail elsewhere [11]. The recorded measures include the time and place of examination, height, weight, educational level, and intelligence test score. See Table 1 for an overview of the included information presented separately for the LiKO-15 and DiaKO-19 studies.

**Table 1:** Overview of what has been measured in the LiKO-15 and DiaKO-19 studies

	**LiKO-15**	**DiaKO-19**
**Baseline assessment (conscription board examination)**		
Personal identification number (CPR-number)	X	X
Date and place of conscription board assessment	X	X
Date of birth and age at examination	X	X
Intelligence test score (BPP, full score)	X	X
Height (cm) and weight (kg)	X	X
Educational level at time of assessment	X	X
**Follow-up assessment**		
Date, age at follow-up, and re-test interval	X	X
Intelligence test score (BPP, full score and subtest scores)	X	X
Anthropometric data		
- Self-reported data on height and weight	X	X
- Measured height, weight, and waist-circumference (subsample of the DiaKO-19 participants)		X
Sociodemographic factors (Educational level, employment status, and type of residence)	X	X
Social relations (Social network (children, partner, living alone), practical and emotional support, loneliness)	X	X
Lifestyle (Alcohol consumption, dietary habits, tobacco consumption, physical activity, sleep quality, and recreational drug use)	X	X
- More detailed information on alcohol use and misuse and recreational drug use	X	
Health (Self-rated health, disease history, use of medication, and Major Depression Inventory)	X	X
- Detailed information on concussions	X	X
- Detailed information on cognitive functioning	X	X
- Detailed information on type 2 diabetes		X
- Detailed information on depression		X
Personality		
- BFI-10	X	
- BFI, subscales of neuroticism and conscientiousness		X
State of mind at the follow-up examination (Recent intake of drugs or medicine and general state of mind at the follow-up examination)	X	X
Motivation at conscription board cognitive ability testing (The extent to which the individual tried his best when completing the BPP)	X	X
**Derived measures**		
Age-related cognitive changes		
- Direct measure derived by subtracting the follow-up BPP-score from the baseline BPP-score	X	X
**Follow-up in national health and administrative registries**		
- Between baseline and follow-up	X	X
- After the follow-up examination	X	X

*Abbreviations*: *LiKO-15* Lifestyle and Cognition Follow-up study 2015, *DiaKO-19* Diabetes and Cognition Follow-up study 2019, *CPR* Central Person Registry, *BPP* Børge Prien’s Prøve (the military intelligence test); BFI: Big Five Inventory

The military intelligence test, Børge Prien’s Prøve (BPP), consists of four subtests, including letter matrices (19 items, 15 min), verbal analogies (24 items, 5 min), number series (17 items, 15 min), and geometric figures (18 items, 10 min) [12]. The participants have a total of 45 minutes to complete the BPP. The items have remained unchanged since 1957, but in 2010 the test was converted from paper-and-pencil to a computer-administered format, which was the format used at the follow-up. Only the BPP total score, corresponding to the number of correct answers summed across the four subtests (range 0–78), is available from the conscription board examinations as subtest-scores were not recorded.

### Follow-up assessment and measures

The DanACo follow-up assessment included the military intelligence test and a questionnaire on sociodemographic factors, lifestyle, and health-related factors. The data collection for LiKO-15 was conducted from September 2015 through June 2017 and the DiaKO-19 data collection was conducted from August 2019 through May 2022. Both data collections were conducted in collaboration with the Selection and Assessment Unit from the Military Recruitment and Career section and were conducted by project staff from the University of Copenhagen in collaboration with military test administrators who were responsible for the administration of the intelligence test. There were two test locations: the University of Copenhagen (LiKO-15: 60% of participants; DiaKO-19: 67% of participants), located in central Copenhagen, and the Selection and Assessment Unit (LiKO-15: 40% of participants; DiaKO-19: 33% of participants) located in a Copenhagen Suburb. The Selection and Assessment Unit was relocated in the summer of 2021 from the Copenhagen Suburb to a central location in Copenhagen.

At the follow-up assessments, the participants received oral information about the project and gave informed consent to participate. A maximum of 24 participants were included per assessment and the intelligence test and questionnaire was completed on computers. In addition, the DiaKO-19 follow-up examination included measurement of height, weight, and waist-circumference.

Due to the COVID-19 pandemic, the DiaKO-19 data collection was interrupted twice (from March 2020 through September 2020 and from December 2020 through April 2021). Following the first interruption, the maximum number of participants per assessment was reduced to 10–12 and the physical measures (height, weight, and waist-circumference) were omitted to ensure sufficient space between participants and personnel.

The military intelligence test was completed on computers at the follow-up assessment and both total and sub-test scores are available, making it possible to derive a direct measure of cognitive changes for the BPP total scores.

The questionnaires administered to the LiKO-15 and DiaKO-19 participants had a high number of identical questions, but they were adapted to the specific study aims and therefore differed with respect to questions related to alcohol consumption, type 2 diabetes and depression. Thus, the LiKO-15 questionnaire had more detailed questions on alcohol consumption, while the DiaKO-19 questionnaire had more detailed information on type-2 diabetes and depression. See Table 1 for a more detailed overview of what has been measured in the two studies.

### Follow-up in national administrative and health registers

In Denmark, several health and administrative registers with complete registration are available for research. The DanACo cohort has been linked to high quality information from registries to obtain life course information on health (e.g. hospital diagnoses and prescribed medications) and on social and economic factors (e.g. income and social benefits) for the period between baseline and follow-up as well as after the follow-up assessments. As an example, information on date and diagnosis from admissions to a psychiatric ward is available from 1969 [13] (age of cohort members: 7–20 years old), and for somatic wards the information is available from 1977 [14] (age of cohort members: 16–37 years). Examples of planned uses of information from registers are to identify potential register-based predictors of cognitive decline (e.g. specific psychiatric or somatic disorders or indicators of labour market affiliation) and furthermore to use registry-data to describe differences between participants and non-participants. And future applications of registry-data would include to investigate the influence of cognitive decline on for example later psychiatric and somatic co-morbidities.

## Utility and discussion

Our research team plans several analyses using the DanACo cohort. Numerous opportunities for data analysis exist due to the comprehensive survey and registry-data available. Consequently, the DanACo research team welcomes collaboration. For more information see the section on “Availability of data and materials”.

### Strengths and weaknesses

Compared with other cognitive aging cohorts, the DanACo cohort has several strengths. First of all, the same test of intelligence was used to assess cognitive ability in both young adulthood and late midlife, and as a result the retest interval is longer (ranging from 26 to 55 years) than in most other studies of cognitive decline [2]. This is important because there is evidence suggesting that long follow-up is essential for identifying predictors of cognitive decline [6]. Another strength is the comprehensive cognitive assessment, which contrasts with studies using screening instruments or tests limited to specific cognitive functions. The military intelligence test (BPP) provides a global measure of cognitive ability without ceiling or floor effects and has been shown to correlate with both demographic and health-related variables [12]. Finally, one of the major strengths of the DanACo cohort is the availability of both comprehensive questionnaire data and prospectively collected information from national registries covering almost the entire period from baseline to follow-up.

As the DanACo cohort was established by combining two separate follow-up studies we conducted a series of analyses to evaluate potential differences between the LiKO-15 and DiaKO-19 study participants. The participants of the two studies were invited according to different criteria, primarily related to the higher proportion of participants with psychiatric disorders in the LiKO-15 study. Consequently, four subsamples of LiKO-15 and DiaKO-19 participants were defined based on whether or not they had a history of psychiatric admissions and the baseline (age, educational level, BPP score) and follow-up (age, retest interval, BPP score) characteristics as well as the difference in BPP scores between baseline and follow-up and the retest correlation were compared across subsamples (see Table A1 in Additional file 1). The lack of significant differences in the baseline mean BPP scores and the very small differences for education and other young adult characteristics suggest that data from the two cohorts can be combined and analyzed as one sample.

Moreover, to assess whether the measurement characteristics of the follow-up BPP were comparable in the LiKO-15 and DiaKO-19 cohorts, we have conducted confirmatory factor analyses (see Table A2 in Additional file 1). The results show that these characteristics safely can be assumed to be essentially the same in the LiKO-15 and DiaKO-19 subsamples, which is assuring when combining the two studies. A potential weakness of the DanACo cohort is the fact that subtest scores were not available for the baseline BPP, why it is not possible to analyse direct measures of decline on the individual subtest scores. Moreover, it is not possible to do confirmatory factor analyses to assess whether measurement invariance can be assumed when the LiKO-15 and DiaKO-19 baseline and follow-up BPP scores are compared. However, we have compared the measurement characteristics of the follow-up BPP from the two studies with the characteristics from a principal component analysis conducted by Hartmann & Teasdale (2004) [15] of BPP subtest scores of 6,757 young men (aged 18–19 years) assessed at the conscription board in 1987. This comparison suggested that the measurement characteristics were essentially the same in the younger sample and in the LiKO-15 and DiaKO-19 samples. Finally, a potential weakness of the DanACo cohort is the change from paper-and-pencil administration (at baseline) to computer-based administration (at follow-up) of the BPP, as there is evidence that the computer-administered version may be slightly more difficult to complete [16]. However, this general factor would presumably have little influence on individual differences in cognitive decline.

The evaluation of the characteristics of the participants and the measurement characteristics of the follow-up BPP for the LiKO-15 and DiaKO-19 studies confirms that the two study samples are sufficiently homogenous to combine them into one cohort. With the total sample of more than 5000 men, it will be possible to achieve sufficient statistical power to identify even relatively weak predictors of cognitive decline. In the DanACo cohort, the standard deviation (SD) of change scores between baseline and follow-up IQ is 9.67, and assuming a group difference in cognitive changes of 0.25 SD, corresponding to a difference of 2.4 IQ points, power will be close to 1.00 regardless of whether we are assuming equal sized groups or with a ratio between exposed and unexposed of 0.33 or 0.18. Assuming a group difference in cognitive changes of 0.10 SD, corresponding to a difference of 1 IQ point, power will be 0.95 if we are assuming equal sized groups, 0.87 if the ratio between exposed and unexposed is 0.33, and 0.73 if the ratio is 0.18.

### Generalizability

The most important weakness of the cohort is the low participation rate as it may limit generalizability of results. To evaluate the generalizability, the baseline characteristics of participants and non-participants were analyzed and the characteristics are presented in Table 2 for the DanACo cohort and separately for the LiKO-15 and DiaKO-19 studies. The mean birth year was comparable among participants and non-participants. Comparing baseline characteristics, the participants were slightly older at conscription, had higher mean intelligence test score, higher education, were slightly taller and weighed slightly more compared to non-participants. Similar tendencies were found for the LiKO-15 and DiaKO-19 studies. Based on information from hospital registers for the time between baseline and follow-up, the DanACo participants had lower prevalence of admissions to a psychiatric ward and lower Charlson Comorbidity Index [17] score (indicating a lower degree of somatic co-morbidity) compared to non-participants. Comparing the LiKO-15 and DiaKO-19 study samples, the proportion of men with a psychiatric admission was significantly higher in the LiKO-15 study both among participants and non-participants. The large proportion of men with prior psychiatric admissions could potentially be problematic for some analyses of cognitive decline. As mentioned, we have performed analyses comparing those with and without psychiatric admissions (for more details see Table A1 in Additional file 1) and these revealed that those with prior psychiatric admissions had a lower mean baseline and follow-up BPP score. However, the mean BPP difference and retest correlation were not significantly different from those with no psychiatric admissions suggesting that the two samples are comparable. Depending on the research question, the participants with a psychiatric history can thus be included in the analyses, analyzed as a separate subsample or excluded from the analyses. Another potential problem with the generalizability in DanACo is underlined by the substantially higher mean baseline intelligence test scores and smaller standard deviations among participants than non-participants. Moreover, the high mean intelligence test scores of the participants may be problematic if effects of some predictors of cognitive decline are limited to low ability levels, and small variance may dilute statistical power. Finally, it is a limitation of the cohort that it only consists of men. The available literature on sex differences in late-life cognitive functioning have shown mixed results depending on the specific cognitive functions measured. Some studies have found higher initial scores on most types of cognitive tests for women except for tests assessing visuospatial ability [18, 19]. Thus, a large European study found that the advantage in cognitive scores depended on region with females having higher scores in Northern Europe but lower scores in Southern Europe [20] whereas previous studies of Danish cohorts have found consistent sex differences with higher performance among men [21, 22]. Most notably, the intelligence test included in the study on the Copenhagen Aging and Midlife Biobank [21] contains subtests that are very similar to two of the BPP subtests. Only few studies have investigated sex differences in cognitive trajectories in old age and the findings are mixed with some studies showing steeper cognitive decline among women [18, 20], others showing steeper decline among men [19, 22], and some showing no significant difference in decline between men and women [6]. It is worth noting that sex differences in late-life cognitive function and in rate of change will not necessarily translate into sex differences in associations between risk factors and cognitive decline. However, we acknowledge that generalisations to women of results based on the DanACo cohort should only be done with great caution and reservation.

**Table 2:** Characteristics of participants and non-participants in the DanACo cohort and the LiKO-15 and DiaKO-19 studies

	**DanACo cohort**			**LiKO-15 study**			**DiaKO-19 study**		
	Participants	Non-participants	*P*-value	Participants	Non-participants	*P*-value	Participants	Non-participants	*P*-value
Total, *n*(%)	5340 (14.3)	32,104* (85.7)		2611 (13.1)	17,277 (86.9)		2729 (12.8)	18,632 (87.2)	
Birth year, Mean(SD)	1954.1 (3.2)	1954.6 (3.2)	< 0.001	1954.4 (3.2)	1955.0 (3.2)	< 0.001	1953.9 (3.1)	1954.3 (3.1)	< 0.001
**Conscription board examination**									
Age, Mean(SD)	20.4 (2.1)	20.0 (1.8)	< 0.001	20.3 (2.0)	19.8 (1.7)	< 0.001	20.5 (2.2)	20.0 (1.9)	< 0.001
BPP-score, Mean(SD)	46.6 (9.5)	39.8 (11.3)	< 0.001	46.2 (9.8)	38.9 (11.4)	< 0.001	47.0 (9.3)	40.6 (11.1)	< 0.001
Educational level, *n*(%)			< 0.001			< 0.001			< 0.001
Low	1492 (28.0)	15,965 (49.8)		782 (30.0)	9017 (52.2)		710 (26.0)	8904 (47.9)	
Medium	1784 (33.5)	9118 (28.4)		871 (33.5)	4856 (28.1)		913 (33.5)	5319 (28.6)	
High	2053 (38.5)	6976 (21.8)		950 (36.5)	3392 (19.7)		1103 (40.5)	4370 (23.5)	
Height, Mean(SD)	179.4 (6.7)	178.5 (7.8)	< 0.001	179.3 (7.1)	178.5 (8.6)	< 0.001	179.5 (6.4)	178.5 (6.6)	< 0.001
Weight, Mean(SD)	69.6 (9.2)	69.1 (11.5)	< 0.001	69.0 (8.9)	68.5 (12.6)	0.012	70.3 (9.5)	69.5 (10.0)	< 0.001
**Information from hospital registers**									
Admission to a psychiatric ward, *n*(%)	112501 (21.1)	8814 (27.5)	< 0.001	863 (33.1)	8068 (46.7)	< 0.001	262 (9.6)	3007 (16.1)	< 0.001
Charlson Comorbidity Index score, Mean(SD)	1.0 (1.7)	1.3 (2.0)	< 0.001	0.9 (1.6)	1.3 (2.1)	< 0.001	1.1 (1.7)	1.3 (1.9)	< 0.001

*Abbreviations*: *DanACo* Danish Aging and Cognition cohort, *LiKO-15* Lifestyle and Cognition Follow-up study 2015, *DiaKO-19* Diabetes and Cognition Follow-up study 2019, *SD* Standard deviation, *BPP* Børge Prien’s Prøve (the military intelligence test)

*3,805 non-participants from the LiKO-15 study were also invited to the DiaKO-19 study. Consequently, the total number of non-participants in the DanACo cohort (*n* = 37,444) is equal to the combined number of non-participants from the LiKO-15 and DiaKO-19 studies (35,909) minus the 3,805 participants that were invited twice

### Published results

Several papers have been published on the LiKO-15 study [23–27], and a few of the main findings will be highlighted. Grønkjær et al. (2019a) [23] found that weekly heavy alcohol consumption (> 28 units per week) averaged over adult-life and accumulated weekly extreme binge drinking (≥ 10 units on the same occasion) was associated with larger age-related cognitive decline. Another study by Grønkjær et al. (2019b) showed that men with a hospital diagnosis of an alcohol-related disorder experienced greater cognitive decline than men without such a diagnosis [24]. Finally, on a subsample of LiKO-15 participants comprising men without psychiatric hospital diagnoses, Grønkjær et al. (2019c) have shown that more education is associated with less cognitive decline in men with low or average cognitive ability in young adulthood, but not in men with high cognitive ability [25], and Wimmelmann et al. (2021) found that young adult intelligence and education was associated with changes in BMI from age 18 to age 61 with education being the stronger predictor [26]. And finally, a study by Michelsen et al. (2022) investigating trajectories of alcohol consumption found that the majority of Danish men drink moderately in the period from young adulthood to late midlife, and deviance from this ‘normal’ moderate consumption trajectory is associated with less favorable social, psychological, lifestyle and health characteristics [27].

There are no published findings from the DiaKO-19 study yet; however, several manuscripts using the study are in process at the present time.

### Plans for future development

The DanACo research team is considering a second follow-up assessment including the military intelligence test (BPP) and questionnaire data. The aim is for the follow-up to be initiated in 2025/26, which is approximately 10 years after the first DanACo participants were tested.

## Conclusion

The unique design of the DanACo cohort is based on data from the mandatory conscription board examinations as baseline and a follow-up examination in late mid-life including the same military intelligence test. This has resulted in a total sample of more than 5000 men with a comprehensive measure of global intelligence measured in both young adulthood and late midlife as well as comprehensive data from a follow-up questionnaire and prospectively collected life-course data from registries. With the long retest interval of more than 40 years and the substantial size of the cohort ensuring sufficient statistical power, we thus believe that the DanACo cohort has some major strengths compared to existing cognitive aging cohorts.

### Supplementary Information

**Additional file 1.** The Danish Aging and Cognition Cohort (DanACo).docx”.

## Data Availability

The data that support the findings of this study are available from the authors but restrictions apply to the availability of these data. For the current study the data were used under license from the data inspection authorities at University of Copenhagen, and so are not publicly available. However, data are available from the authors upon reasonable request and with permission from relevant authorities. Queries regarding data access and more information about the cohort can be directed to Gunhild Tidemann Okholm [guch@sund.ku.dk or gunhild.tidemann.okholm@regionh.dk] or to the DanACo research team [bfh-fp-ckff-danaco@regionh.dk].

## Abbreviations

BPP: Børge Priens Prøve, the intelligence test developed and used by the Danish Defence
DanACo: Danish Aging and Cognition
DiaKO-19: Diabetes and Cognition Follow-up study 2019
LiKO-15: Lifestyle and Cognition Follow-up study 2015
