# Supplementary material for: The Danish Aging and Cognition (DanACo) cohort
Source: BMC Geriatr. 2024 Mar 7;24:238. doi: 10.1186/s12877-024-04841-5 (PMC10921587; doi:10.1186/s12877-024-04841-5)
Supplement: Supplementary file 1 — Additional file 1. The Danish Aging and Cognition Cohort (DanACo).docx”. [file 12877_2024_4841_MOESM1_ESM.docx]

# Additional material

Title: The Danish Aging and Cognition Cohort (DanACo)

**Table A1. Characteristics of the LiKO-15 and DiaKO-19 subsamples and combined samples**

| Variable | LiKO-15  non-psychiatric  subsample | DiaKO-19  non-psychiatric  subsample | Combined  psychiatric subsample  (LiKO-15 +DiaKO-19) | Combined non-psychiatric subsample  (LiKO-15+DiaKO-19) |
| --- | --- | --- | --- | --- |
| *N* | 1677 | 2410 | 1096 | 4087 |
|  | **Mean (SD)** | **Mean (SD)** | **Mean (SD)** | **Mean (SD)** |
| Birth year | 1954.3 (3.2) | 1953.9 (3.1) | 1954.4 (3.2) | 1954.0 (3.2) |
| **Age** |  |  |  |  |
| Baseline | 20.3 (2.1) | 20.5 (2.2) | 20.2 (2.0) | 20.4 (2.1) |
| Follow-up | 61.5 (3.3) | 67.0 (3.2) | 62.9 (3.7) | 64.8 (4.2) |
| Retest interval | 41.2 (3.3) | 46.6 (3.6) | 42.7 (3.9) | 44.4 (4.4) |
| **School education** |  |  |  |  |
| Low (*n*, %) | 470 (28.1) | 622(25.8) | 357 (32.7) | 1092 (26.8) |
| Medium (*n*, %) | 558 (33.3) | 795 (33.0) | 371 (34.0) | 1353 (33.2) |
| High (*n*, %) | 646 (38.6) | 990 (41.1) | 363 (33.3) | 1636 (40.1) |
| **Education years** | 13.7 (2.4) | 13.9 (2.5) | 13.3 (2.6) | 13.8 (2.5) |
| **BPP** |  |  |  |  |
| Baseline BPP | 47.0 (9.3) | 47.3 (9.3) | 44.7 (10.4) | 47.2 (9.3) |
| Follow-up BPP | 44.0 (8.8) | 42.7 (8.9) | 40.5 (10.2) | 43.2 (8.9) |
| Difference in BPP* | -3.0 (5.6) | -4.5 (6.1) | -4.1 (6.8) | -3.9 (6.0) |
| Retest correlation (r, 95%CI) | 0.80 (0.79-0.82) | 0.77 (0.76-0.79) | 0.78 (0.76-0.80) | 0.79 (0.77-0.80) |

Abbreviations: LiKO-15: Lifestyle and Cognition Follow-up study 2015; DiaKO-19: Diabetes and Cognition Follow-up study 2019; SD: Standard deviation; BPP = Børge Prien’s Prøve (the military intelligence test); CI: confidence interval.

*The minor discrepancies between the reported “Difference in BPP” and the difference between the reported Baseline and Follow-up BPPs are due to rounding of figures.

**Comparison of subsamples and combined samples**

As described, the DanACo cohort was established by combining two separate cognitive follow-up studies and the participants of these two studies were invited according to different criteria. The proportion of participants having been admitted to a psychiatric department prior to the follow-up examination is 3.5 times higher in the LiKO-15 study (33.5 %) than in the DiaKO-19 study (9.4%) (see Table 2). Given the well-documented association between psychiatric history and lower intelligence (1–3), we thus decided to define two separate subsamples and two combined subsamples of LiKO-15 and DiaKO-19 participants based on whether or not they had a history of psychiatric admissions: the LiKO-15 and DiaKO-19 non-psychiatric subsamples comprising participants with no history of psychiatric admissions, a combined (LiKO-15+DiaKO-19) psychiatric subsample, comprising all participants with a history of psychiatric admissions, and a combined (LiKO-15+DiaKO-19) non-psychiatric subsample. The baseline and follow-up characteristics of the four samples are presented in Table A1 to evaluate potential differences between samples. The p-value for the overall F test comparing the LiKO-15 and DiaKO-19 non-psychiatric subsamples with the combined psychiatric subsample was significant for all variables, and consequently contrasts between the LiKO-15 and DiaKO-19 non-psychiatric subsamples and the combined psychiatric sample were evaluated. The analyses showed that the DiaKO-19 non-psychiatric subsample differed significantly from the LiKO-15 subsample and from the combined psychiatric subsample with respect to birth year and age at baseline while all three of the samples differed significantly with respect to follow-up age, retest interval between the baseline and follow-up assessment, and years of education (in spite of the small difference between the two subsamples without psychiatric history). It is important to note that except for follow-up age and the retest-interval, the differences in means among these three samples were relatively small and potentially only significant because of the large sample sizes.

With respect to the intelligence test scores (BPP scores), the younger LiKO-15 non-psychiatric subsample performed better at the follow-up than the older DiaKO-19 subsample, suggesting significantly more decline in BPP scores in the older DiaKO-19 subsample. In fact, the mean BPP decline of 4.5 in the DiaKO-19 subsample is close to half a standard deviation of the baseline BPP in both the LiKO-15 and DiaKO-19 subsamples. To test for significant differences among the LiKO-15 and DiaKO-19 subsamples and the combined psychiatric subsample, a mixed model including 2 x 2 fixed factors (group x time) and participant as a random effect to take the correlation between repeated BPP tests into account was analyzed (data not shown). This model showed no significant group differences for baseline BPP, but a significant group difference was found for the follow-up BPP scores and for the differences between baseline and follow-up BPP scores. Using a similar model, the combined non-psychiatric sample was compared with the combined psychiatric subsample and showed significantly lower baseline and follow-up BPP scores in the psychiatric subsample, but the small difference in mean decline scores was not significant. The lower baseline BPP score among men with previous psychiatric admissions is in line with several previous studies(1,2).

Table A1 also shows substantial retest correlations for all four subsamples. There is a small, but significant difference in the retest correlations for the LiKO-15 and DiaKO-19 subsamples. The smaller retest correlation in the DiaKO-19 subsample most likely reflects larger individual differences in decline and the longer retest interval for this group. Notably, the mean BPP difference between baseline and follow-up and the retest correlation of the psychiatric subsample were not significantly different from those of the combined non-psychiatric sample, suggesting that the two samples are comparable.

Since the participants in the two studies appeared before a conscription board at about the same age and the two subsamples without psychiatric history obtained similar mean BPP scores, the differences in mean follow-up BPP and decline are likely to reflect the longer follow-up interval and the higher age at follow-up in the DiaKO-19 study. The latter two variables were fixed by design, and the most important finding in relation to combing these two subsamples is the lack of significant differences in the baseline mean BPP scores. This result and the small differences for education and other young adult characteristics suggest that data from the two studies can be combined and analyzed as one sample. The large proportion of men with prior psychiatric admissions could be problematic for some analyses of cognitive decline. Depending on the research question, the participants with psychiatric history can thus either be included in the analyses, analyzed as a separate subsample, or excluded from the analyses.

**Table A2. Confirmatory multi group factor analysis: Coefficients and reliability**

| Variable | Fixed coefficients | LiKO-15  Mean (SD) | DiaKO-19  Mean (SD) | Principal  components |
| --- | --- | --- | --- | --- |
| *N* | 3960 | 1531 | 2429 | 3960 |
| Letter Matrices | 0.799 (0.771-0.827) | 0.798 (0.767-0.829) | 0.800 (0.770-0.829) | 0.816 |
| Verbal Analogies | 0.773 (0.744-0.801) | 0.775 (0.743-0.807) | 0.771 (0.742-0.801) | 0.800 |
| Number Series | 0.710 (0.685-0.736) | 0.701 (0.670-0.733) | 0.716 (0.689-0.744) | 0.812 |
| Geometric Figures | 0.531 (0.504-0.558) | 0.533 (0.494-0.571) | 0.529 (0.497-0.562) | 0.686 |
| P value model | 0.802 |  | 0.615 |  |
| RMSEA | 0.000 (0.000-0.015) |  | 0.000 (0.000-0.023) |  |
| CFI | 1.000 |  | 1.000 |  |
| Omega reliability | 0.815 (0.800-0.830) | 0.818 (0.792-0.843) | 0.813 (0.794-0.832) |  |

Abbreviations: LiKO-15: Lifestyle and Cognition Follow-up study 2015; DiaKO-19: Diabetes and Cognition Follow-up study 2019; SD: Standard deviation; BPP = Børge Prien’s Prøve (the military intelligence test).

**Comparison of the psychometric characteristics of BPP across subsamples**

With regard to the psychometric characteristics of the BPP, Grønkjær et al. (2019a) (4) conducted confirmatory factor analysis of the follow-up data of a subsample of LiKO-15 participants who had never been admitted to a psychiatric ward at baseline. To assess whether the psychometric characteristics of the BPP were similar in LiKO-15 and DiaKO-19, a subsample of DiaKO-19 participants who at baseline had never been admitted to a psychiatric ward was defined and a multi-group analysis was conducted using the subtest scores from the follow-up assessment. The results are presented in Table A2, which shows standardized loadings for a one-factor model allowing loading differences between the two subsamples and a model with loadings constrained to be equal in the two subsamples. Like Grønkjær et al. (2019a)(4), we found that the best fitting one-factor model allowed for correlated error terms for letter matrices and verbal analogies. The analyses revealed almost identical loadings in the two subsamples even in a model allowing for loading differences between the subsamples (Table A2). Based on these results, we conclude that the measurement characteristics of the BPP can be assumed to be essentially the same in the LiKO-15 and DiaKO-19 subsamples. Based on the one-factor model, the omega measure of reliability (5) was calculated for the LiKO-15 and DiaKO-19 subsamples and the combined total sample. All three estimates were very close in the 0.81 to 0.82 range, suggesting higher reliability than the 0.74/0.77 retest reliability reported by Teadale et al. (2011) (6).

1. Urfer-Parnas A, Lykke Mortensen E, Saebye D, Parnas J. Pre-morbid IQ in mental disorders: a Danish draft-board study of 7486 psychiatric patients. Psychol Med. 2010 Apr;40(4):547–56.

2. Mortensen EL, Sorensen HJ, Jensen HH, Reinisch JM, Mednick SA. IQ and mental disorder in young men. Br J Psychiatry. 2005 Nov;187:407–15.

3. Christensen GT, Rozing MP, Mortensen EL, Christensen K, Osler M. Young adult cognitive ability and subsequent major depression in a cohort of 666,804 Danish men. Journal of Affective Disorders. 2018 Aug;235:162–7.

4. Grønkjær M, Osler M, Flensborg-Madsen T, Sørensen HJ, Mortensen EL. Associations between education and age-related cognitive changes from early adulthood to late midlife. Psychology and Aging. 2019;34:177–86.

5. McDonald RP. Test theory: A unified treatment. Mahwah, NJ, US: Lawrence Erlbaum Associates Publishers; 1999. xi, 485 p. (Test theory: A unified treatment).

6. Teasdale TW, Hartmann PV, Pedersen CH, Bertelsen M. The reliability and validity of the Danish Draft Board Cognitive Ability Test: Borge Prien’s Prove. Scand J Psychol. 2011 Apr;52(2):126–30.
